# The role of pro-opiomelanocortin in the ACTH–cortisol dissociation of sepsis

**DOI:** 10.1186/s13054-021-03475-y

**Published:** 2021-02-16

**Authors:** Arno Téblick, Sarah Vander Perre, Lies Pauwels, Sarah Derde, Tim Van Oudenhove, Lies Langouche, Greet Van den Berghe

**Affiliations:** Clinical Division and Laboratory of Intensive Care Medicine, Department of Cellular and Molecular Medicine, KU Leuven, Herestraat 49, 3000 Leuven, Belgium

**Keywords:** Sepsis, Pituitary, Adrenal, Adrenocorticotropic hormone, Pro-opiomelanocortin, Glucocorticoid receptor

## Abstract

**Background:**

Sepsis is typically hallmarked by high plasma (free) cortisol and suppressed cortisol breakdown, while plasma adrenocorticotropic hormone (ACTH) is not increased, referred to as ‘ACTH–cortisol dissociation.’ We hypothesized that sepsis acutely activates the hypothalamus to generate, via corticotropin-releasing hormone (CRH) and vasopressin (AVP), ACTH-induced hypercortisolemia. Thereafter, via increased availability of free cortisol, of which breakdown is reduced, feedback inhibition at the pituitary level interferes with normal processing of pro-opiomelanocortin (POMC) into ACTH, explaining the ACTH–cortisol dissociation. We further hypothesized that, in this constellation, POMC leaches into the circulation and can contribute to adrenocortical steroidogenesis.

**Methods:**

In two human studies of acute (ICU admission to day 7, *N* = 71) and prolonged (from ICU day 7 until recovery; *N* = 65) sepsis-induced critical illness, POMC plasma concentrations were quantified in relation to plasma ACTH and cortisol. In a mouse study of acute (1 day), subacute (3 and 5 days) and prolonged (7 days) fluid-resuscitated, antibiotic-treated sepsis (*N* = 123), we further documented alterations in hypothalamic CRH and AVP, plasma and pituitary POMC and its glucocorticoid-receptor-regulated processing into ACTH, as well as adrenal cortex integrity and steroidogenesis markers.

**Results:**

The two human studies revealed several-fold elevated plasma concentrations of the ACTH precursor POMC from the acute to the prolonged phase of sepsis and upon recovery (all *p* < 0.0001), coinciding with the known ACTH–cortisol dissociation. Elevated plasma POMC and ACTH–corticosterone dissociation were confirmed in the mouse model. In mice, sepsis acutely increased hypothalamic mRNA of CRH (*p* = 0.04) and AVP (*p* = 0.03) which subsequently normalized. From 3 days onward, pituitary expression of CRH receptor and AVP receptor was increased. From acute throughout prolonged sepsis, pituitary POMC mRNA was always elevated (all *p* < 0.05). In contrast, markers of POMC processing into ACTH and of ACTH secretion, negatively regulated by glucocorticoid receptor ligand binding, were suppressed at all time points (all *p* ≤ 0.05). Distorted adrenocortical structure (*p* < 0.05) and lipid depletion (*p* < 0.05) were present, while most markers of adrenocortical steroidogenic activity were increased at all time points (all *p* < 0.05).

**Conclusion:**

Together, these findings suggest that increased circulating POMC, through CRH/AVP-driven POMC expression and impaired processing into ACTH, could represent a new piece in the puzzling ACTH–cortisol dissociation.

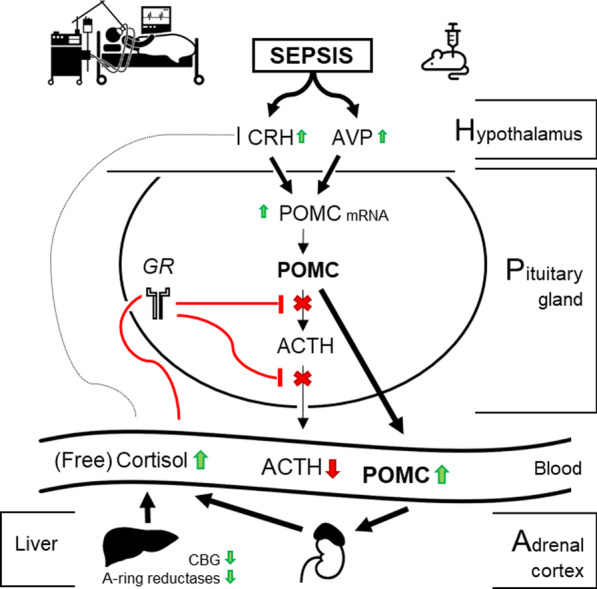

## Introduction

Patients suffering from critical illnesses, which can be evoked by sepsis, major trauma, extensive burn injuries or surgery, typically present with high plasma concentrations of total and even more so of free cortisol [1, 2]. The increased systemic cortisol availability during critical illness is crucial for survival as it plays a key role in providing essential energy substrates and in regulating the immune and hemodynamic responses necessary for restoring homeostasis [3–5]. Both very high and very low levels of systemic cortisol have been associated with poor outcome, underlining the importance of a thorough understanding of this response [2].

Hypercortisolism of critical illness has long been assumed to be exclusively brought about by a centrally activated hypothalamic–pituitary–adrenocortical (HPA) axis. This centrally activated HPA axis implies activated hypothalamic corticotropin-releasing hormone (CRH) and vasopressin (AVP) expression, in turn driving pituitary production of the 31-kDa large precursor poly-peptide hormone, pro-opiomelanocortin (POMC). Newly synthesized POMC proteins are predominantly sorted in dense secretory core granules within the corticotropic cells and are subsequently cleaved into adrenocorticotropic hormone (ACTH) by proprotein convertase 1 (PC1/3) [6–8]. Subsequently, ACTH is released in the systemic circulation and rapidly activates the adrenal cortex to synthesize and release cortisol [7]. However, the hypercortisolism of critical illness is not accompanied by elevated plasma ACTH. This has been referred to as ‘ACTH–cortisol dissociation’ [1, 9].

Over the last decade, it has been shown that the rise in systemic cortisol availability during critical illness is to a large extent explained by suppression of the cortisol-binding proteins, increasing the free fraction of cortisol in the circulation, and by suppression of cortisol breakdown in liver and kidney [1, 10]. Whether the low plasma ACTH is the consequence of glucocorticoid-receptor (GR)-mediated feed-back inhibition exerted by high circulating cortisol driven through these peripheral mechanisms remains debated [2, 9]. In addition, the site of such feed-back inhibition (hypothalamus, pituitary or both), if present, and the affected pathways remain incompletely understood.

In the subacute and chronic phases of illness, incremental ACTH responses to a bolus injection of CRH were shown to be suppressed [11]. Upon recovery, one week after intensive care discharge, rebound rises in plasma ACTH and cortisol to supra-normal levels have been reported [10]. These data suggest the possibility of a centrally suppressed adrenocortical function when critical illness lingers. However, the finding that ACTH is not fully suppressed while circulating free cortisol is substantially elevated, suggests an ongoing central stimulation [2, 9]. Indeed, while ACTH was low, cortisol production rates documented via tracer technology were found to be doubled as compared with healthy subjects [1]. Hence, adrenocortical stimulation not exerted by ACTH may contribute to steroidogenesis in these patients, while elevated free cortisol could exert central feed-back inhibition.

We hypothesized that sepsis-induced critical illness, further referred to as ‘sepsis,’ indeed immediately and continuously activates the hypothalamus to generate, via CRH and AVP, ACTH-induced hypercortisolism, but as soon as free cortisol is elevated, feedback inhibition at the pituitary level interferes with normal processing of POMC into ACTH, explaining the typical ACTH–cortisol dissociation. In this constellation, unprocessed POMC could leach from the pituitary into the systemic circulation, which could in theory stimulate the adrenal cortex [12, 13]. To test this hypothesis, we first documented plasma concentrations of POMC in relation to ACTH and cortisol in acute and prolonged human critical illness evoked by sepsis. Subsequently, we performed a study in septic mice to document the hypothesized alterations within the hypothalamus, the pituitary and the adrenal cortex in relation to duration of illness.

Part of these results has been previously reported in the form of an abstract [14].

## Methods

### Human studies of critically ill patients with sepsis

Two human studies were performed to document the plasma POMC concentration time course during sepsis-induced critical illness and in the recovery phase, in relation to ACTH and cortisol. Human study 1 focused on ICU patients during the first week in ICU. We selected, from a previous study [15], all available patients who suffered from sepsis at study inclusion (*n* = 51), according to the 1992 Sepsis-2 criteria [16], and who did not meet exclusion criteria and 20 demographically matched overnight-fasted healthy controls. Exclusions criteria were pre-admission risks for HPA axis dysfunction, which comprised chronic treatment with glucocorticoids, or anti-steroid chemotherapy within the last 3 months, steroid treatment in the hours preceding ICU admission (e.g., during surgery or in the emergency room) or other drugs predisposing to adrenal insufficiency (phenytoin, rifampicin, glitazones, imipramine, phenothiazine, phenobarbital). In this study, plasma samples were collected daily throughout the first week in ICU. Human study 2 focused on the prolonged phase of critical illness, beyond 1 week in the ICU until recovery on a regular ward [10]. From this study, all available patients who suffered from sepsis at inclusion (*n* = 45) with an ICU stay of at least 4 weeks and 20 demographically matched healthy controls were included, with similar exclusion criteria as for human study 1. In this study, plasma samples were collected weekly, beyond the first week in ICU until ICU day 49 and 1 week after ICU discharge. Baseline characteristics are described in Table 1. Written informed consent was obtained from all patients and their next of kin and from all healthy volunteers. The study protocols and consent forms were approved by the Institutional Ethical Review Board (ML4190 and ML11107). Total plasma cortisol concentrations were quantified with the use of a radioimmunoassay (Immunotech), plasma ACTH concentrations with the use of an immunoradiometric assay (Brahms Diagnostics) and plasma POMC concentrations with the use of an enzyme-linked immunosorbent assay (MyBioSource Inc.), with details provided in Additional file 1.

**Table 1:** Baseline characteristics and demographics of human study 1 and human study 2

	Human study 1		*P* value	Human study 2		*P* value
	Patients *n* = 51	Controls *n* = 20		Patients *n* = 47	Controls *n* = 20	
Gender—male, *n* (%)	33 (68%)	11 (55%)	.45	37 (79%)	14 (70%)	.44
Age—y, mean (SD)	60.0 ± 5.1	58.5 ± 15.8	.55	63.3 ± 13.7	64.1 ± 10.9	.80
BMI^a^—kg/m^2^, mean (SD)	26.1 ± 4.6	24.3 ± 3.0	.06	27.5 ± 5.8	26.6 ± 3.0	.40
Diabetes mellitus, *n* (%)	8 (16%)			8 (17%)		
Malignancy, *n* (%)	14 (27%)			9 (19%)		
APACHE II score^b^, mean (SD)	30.3 ± 8.2			32.0 ± 7.4		
Urgent admission, *n* (%)	44 (86%)			41 (87%)		
Sepsis^c^, *n* (%)	51 (100%)			47 (100%)		
Septic shock^c^, *n* (%)	13 (25%)			38 (81%)		

^a^Body mass index (BMI) is calculated as the weight in kilograms divided by the square of the height in meters

^b^Acute Physiology and Chronic Health Evaluation II (APACHE II) score reflects severity of illness, with higher values indicating more severe illness, and can range from 0 to 7 [33]

^c^Sepsis and septic shock were defined as in Ref. [16]

### Mouse model of sepsis

To study the impact of sepsis on hormonal alterations and possibly involved pathways in the liver, the hypothalamus and the pituitary and the relationship hereof with abnormalities in the adrenal cortex, male, 24-week-old C57BL/6J mice (Janvier SAS) were randomly allocated to a ‘sepsis’ or a ‘healthy control’ group. To assess the impact of duration of sepsis, both the ‘sepsis’ and ‘healthy control’ groups were subdivided into four time-cohort groups with increasing duration of illness (1 day, 3 days, 5 days or 7 days). Animals randomized to the ‘healthy control group’ did not undergo any procedure and were transferred to individual cages and received ad libitum standard chow (ssniff R/M-H, ssniff Spezialdiäten GmbH) and tap water throughout the study period. Animals randomized to ‘sepsis’ were anesthetized, and the left internal jugular vein was cannulated with a catheter, followed by a median laparotomy and cecal ligation and puncture to induce sepsis. During the first 24 h after the procedure, mice were resuscitated with a 4:1 crystalloid/colloid mixture (Plasmalyte, Baxter). Hereafter, septic mice allocated to the 3-day, 5-day or 7-day sepsis groups received intravenous parenteral nutrition (Oliclinomel N7E, Baxter). Septic mice further received twice daily a subcutaneous injection with broad-spectrum antibiotics (imipenem/cilastatin (Aurobindo Pharma)) and opioid analgesics (buprenorphine (Vetergesic)). All animal cages were kept in an animal cabinet under controlled temperature (27 °C) and 12-h light and dark cycles. At the end of the study period, mice received intraperitoneal ketamine/xylazine and were killed with terminal cardiac puncture to collect whole blood samples and tissue samples were collected, snap-frozen and stored at − 80 °C for later analysis. The study was continued until at least 15 surviving animals per study group and per time cohort were reached. This sample size was based on an estimated effect size of 50% increase in plasma corticosterone (CORT), a 15% reduction in CRH expression and a 50% reduction in adrenal lipid content, to be detected with an α-error ≤ 0.05 and > 80% power. The total number of animals per group at the end of the study is as follows: Healthy 1 day: *n* = 15, Healthy 3 days: *n* = 15, Healthy 5 days: *n* = 15, Healthy 7 days: *n* = 16, Sepsis 1 day: *n* = 15, Sepsis 3 days: *n* = 16, Sepsis 5 days: *n* = 16, Sepsis 7 days: *n* = 15. All animals were treated according to the Principles of Laboratory Animal Care (US National Society of Medical Research) and to the European Union Directive 2010/63/EU concerning the welfare of laboratory animals. The study was approved by the Institutional Ethical Committee for Animal Experimentation (P134-2013) and complied with the essential 10 ARRIVE guidelines [17]. Additional information on the animal model is provided in Additional file 1.

### Plasma analyses of HPA hormones and binding proteins

Plasma concentrations of CORT, ACTH and POMC were quantified using commercially available enzyme-linked immunosorbent assays kits (DRG). Plasma cortisol-binding globulin (CBG) was quantified by western blot (Abcam). Plasma albumin was measured with a colorimetric assay (Thermo Scientific). As free CORT measurements were not feasible, levels of free plasma CORT were estimated based on plasma total cortisol, CBG and albumin concentrations with the following calculation [relative plasma total corticosterone—0.85*relative plasma CBG*relative plasma total corticosterone)—(0.10*relative plasma albumin*relative plasma total corticosterone)]. Data are presented as fold difference as compared with the median of the day-1 healthy control mice (arbitrary unit, AU). Additional information on the used assays is provided in Additional file 1.

### *Hypothalamic CRH and AVP expression with *in situ* hybridization*

We performed chromogenic RNAscope in situ hybridization (Advanced Cell Diagnostics) to quantify gene expression of CRH and AVP in the paraventricular nucleus (PVN) of the hypothalamus. RNAscope 2.5 HD Duplex Assay was performed as per the manufacturer’s instructions [18] on slides containing a brain section through the PVN. Images were captured at 40 × magnification using a Leica DM3000 bright-field microscope. Intensities of CRH and of AVP were scored semiquantitatively. A four-level score system was defined as: ‘0’ for absent to low staining, ‘1’ low to moderate staining, ‘2’ high staining and ‘3’ very high staining to saturation of the region of interest. Additional assay details are provided in Additional file 1.

### RNA isolation and reverse transcription polymerase chain reaction analysis

In the pituitary, we quantified gene expression of (1) the ACTH precursor POMC, (2) the main processing enzymes cleaving POMC into smaller fragments: proprotein convertase 1 (PC1/3) and proprotein convertase 2 (PC2), (3) the transcriptional regulators hereof, which are themselves regulated at the mRNA levels by CRH, AVP and/or a GR ligand (CRH receptor (CRH-R), AVP receptor (AVP-R), GR, Nur77 and Tpit), (4) a GR-ligand-binding-stimulated inhibitor of CRH-driven secretion of mature ACTH, Annexin A1, and (5) pro-inflammatory cytokines (tumor necrosis factor alpha (TNF-α) and leukemia inhibitory factor (LIF)). In the liver, we quantified gene expression of the main hepatic CORT-metabolizing enzymes 5α-reductase and 5β-reductase. In the adrenal gland, we quantified gene expression of (1) key regulators of adrenal steroidogenesis (melanocortin receptor 2 (MC2-R) and MC2-R accessory protein (MRAP)), (2) key markers of adrenal steroidogenesis (high-density lipoprotein receptor (HDL-R), low-density lipoprotein receptor (LDL-R), 3-hydroxy-3-methylglutaryl coenzyme A reductase (HMG-CoA reductase), steroidogenic acute regulatory protein (StAR), cholesterol side-chain cleavage (P450scc), steroid 11β-hydroxylase) and (3) markers of inflammation (TNF-α). In brief, RNA was isolated from pituitary, liver and adrenal tissue and reverse-transcribed into complementary DNA (cDNA). Next, cDNA was quantified in real time with the use of commercial TaqMan and SYBR Green assays. Data of gene expression are normalized to Rn18s expression, a stable housekeeping gene, and are presented as fold difference as compared with the median of the respective healthy controls. Additional information on the assays is provided in Additional file 1.

### Protein isolation and immunoblotting

We quantified pituitary protein content of the hormones POMC and ACTH and of the enzyme PC1/3. In brief, proteins were isolated from a whole pituitary homogenate, separated on an SDS–PAGE gel and electroblotted as described in Additional file 1. Data of protein content are presented as fold difference as compared with the median of the healthy controls.

### Structural integrity and quantification of cholesterol ester content of the adrenal cortex

Structural integrity of the adrenal cortex was evaluated on hematoxylin-and-eosin-stained tissue sections of the adrenal gland and scored semiquantitatively. A ‘2’ score was given when the three adrenocortical zones were clearly distinguishable and the fasciculate zone had a normal, radial, cord-like architectural pattern; a ‘1’ score was given when there was a moderate distortion of the adrenocortical zones with or without the presence of tissue edema; a ‘0’ score was given when the three adrenocortical zones were severely distorted with the presence of extensive tissue damage. Adrenocortical cholesterol esters storage was quantified on Oil-Red-O (ORO)-stained tissue sections and analyzed for the relative amount of redness in the adrenal cortex with ImageJ 1.52a. Additional information on the assays is provided in Additional file 1.

### Statistical analysis

Data are presented as box plots with median, interquartile range (25th–75th percentiles) and 10^th^ and 90^th^ percentiles or as mean and standard error of the mean (SEM). Differences between groups were analyzed with the use of Mann–Whitney U, Chi-squared or Fisher exact test, as appropriate. Time-series data from both human studies were analyzed with use of repeated-measures ANOVA, after transformation of the results to obtain a normal distribution. A two-sided *p* value equal to or less than 0.05 was considered statistically significant. No corrections for multiple comparisons were performed. All statistical analyses were done with JMP Pro 14 (SAS Institute Inc.).

## Results

### Human studies of critically ill patients suffering from sepsis

**Fig. 1:**
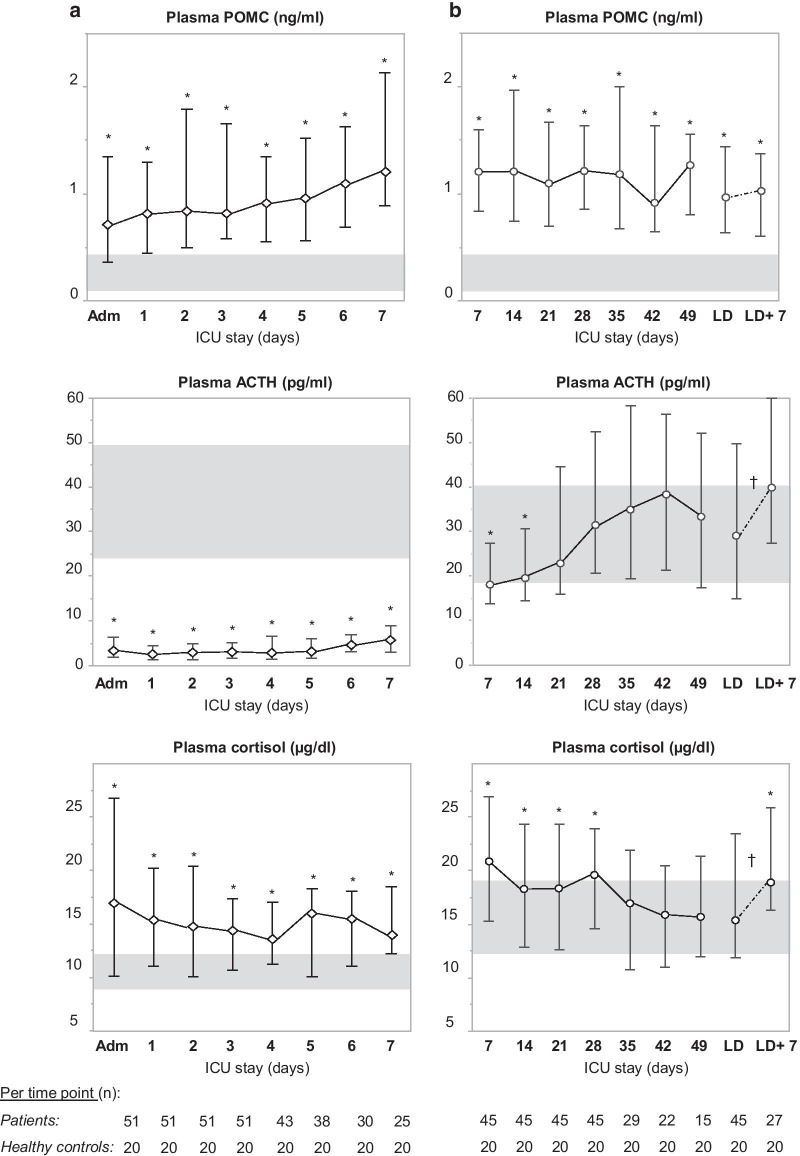
Plasma cortisol, ACTH and POMC concentrations in critically ill patients. **a** During the first week of critical illness (human sepsis study 1). **b** Beyond the first week of critical illness (human sepsis study 2). Top panels display morning plasma POMC concentrations, middle panels morning plasma ACTH concentrations and lower panels morning plasma cortisol concentrations. Diamonds (study 1) and circles (study 2) and whiskers represent median and interquartile ranges of plasma POMC concentrations of critically ill patients per day. The solid line connects the medians of each day. The light gray area represents the interquartile range of morning values in healthy controls (*n* = 20). The dashed line represents the interval between the median concentration of cortisol, ACTH or POMC during the last ICU day and those of the sample taken seven days after ICU discharge. *p < 0.05 between critically ill patients and healthy controls. †*p* < 0.05 between LD and LD + 7 (within-subjects effect of time)

During the first week in ICU, plasma concentrations of ACTH in patients were always lower than those of healthy controls (*p* < 0.0001 for each time point, median across all time points: 3.26 pg/ml (IQR 1.57–5.87) versus 30.04 pg/ml (IQR 24.52–48.92)), whereas plasma concentrations of total cortisol were always higher in patients than normal (*p* < 0.01 for each time point, median across all time points: 14.65 µg/dl (IQR 10.67–19.09) versus 11.22 µg/dl (IQR 8.99–12.04)) (Fig. 1a). In contrast, at ICU admission, patients had higher than normal plasma POMC concentrations as compared with healthy control subjects (*p* < 0.0001, median: 0.72 ng/ml (IQR 0.36–1.35) versus 0.1 ng/ml (IQR 0.1–0.42)). Plasma POMC concentrations of patients further increased over time and were still elevated on ICU day 7 as compared with healthy controls (all *p* < 0.0001, median on day 7: 1.21 ng/ml (IQR 0.89–2.13) versus 0.1 ng/ml (IQR 0.1–0.42)) (Fig. 1a). Prolonged sepsis patients, hospitalized for 4 weeks or more in the ICU, had high plasma concentrations of total cortisol on day 7 (median patients: 20.83 µg/dl (IQR 15.26–26.93) versus healthy controls: 13.75 µg/dl (IQR 12.36–18.99)) in the face of low plasma ACTH concentrations (median patients: 17.88 pg/ml (IQR 13.73–27.41) versus healthy controls: 30.29 pg/ml (IQR 18.70–40.25)), whereas from ICU day 35 and ICU day 21 onwards, plasma concentrations of total cortisol and ACTH, respectively, were no longer different from those in healthy control subjects (Fig. 1b). Upon recovery, as compared with the last ICU day, 7 days after ICU discharge plasma concentrations of total cortisol and ACTH increased (LD to LD + 7, within-subjects effect of time, *p* = 0.001 and *p* = 0.02, respectively), with cortisol concentrations at that time point reaching supra-normal values (*p* = 0.005, median patients: 18.76 µg/dl (IQR 16.31–25.96) versus healthy controls: 13.75 µg/dl (IQR 12.36–18.99)). In contrast, among these long-stay patients, plasma POMC concentrations were higher than normal on day 7 (*p* < 0.0001, median: 1.20 ng/ml (IQR 0.84–1.60) versus 0.1 ng/ml (IQR 0.1–0.41)), remained increased throughout the entire course of illness (all *p* < 0.0001) and were still similarly increased 7 days after ICU discharge (*p* < 0.0001 as compared with healthy controls; *p* = 0.09 for LD to LD + 7 within-patients effect of time) (Fig. 1b).

### Mouse study

#### Plasma concentrations of total and free CORT and of ACTH and its precursor POMC and hepatic expression of the cortisol-metabolizing enzymes 5α-reductase and 5β-reductase in acute and prolonged sepsis-induced critical illness

**Fig. 2:**
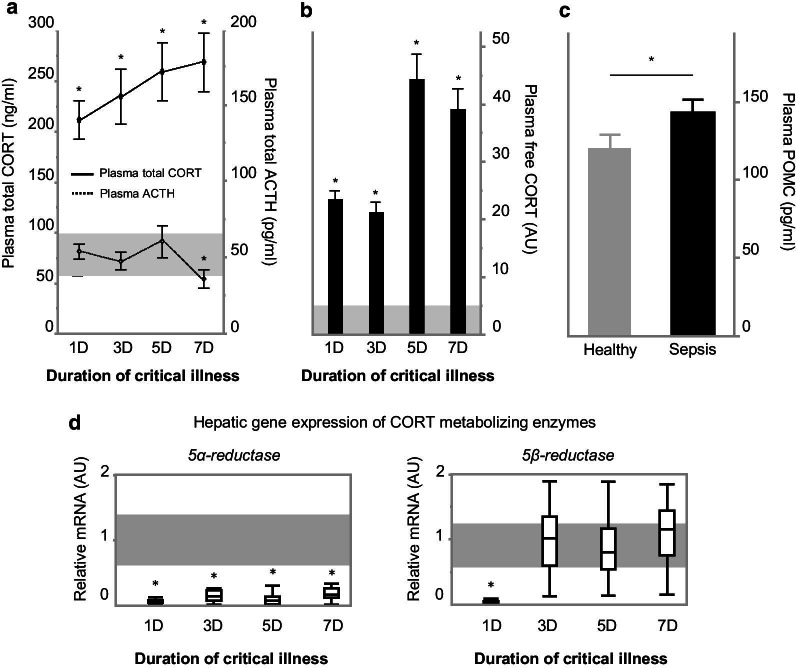
Plasma (free) CORT, ACTH and POMC concentrations and hepatic gene expression of A ring reductases. **a** Diamonds and whiskers represent plasma total corticosterone concentrations (full line, linear scale on the left *Y*-axis, ng/ml) and plasma ACTH concentrations (dashed line, linear scale on the right *Y*-axis, pg/ml). **b** Bars represent estimated free corticosterone levels (linear scale on the right *Y*-axis, AU: fold of healthy control). Shaded gray area represents healthy reference range (mean ± SEM of the healthy control groups over all days). **c** Bars represent plasma POMC concentrations (linear scale on the right *Y*-axis, pg/ml). All data are presented as mean ± SEM. **d** Gene expression of hepatic CORT-metabolizing enzymes 5α- and 5β-reductase. Box-and-whiskers represent median and interquartile ranges (AU: fold of healthy control).*p ≤ 0.05 between sepsis and healthy control mice. AU, arbitrary units; D, days

Plasma total CORT concentrations increased acutely (1-day sepsis group) up to threefold and remained increased over time (3-day, 5-day and 7-day sepsis groups) during sepsis as compared with healthy control mice (*p* < 0.0001 for all time cohorts) (Fig. 2a). Estimated plasma-free CORT concentrations were increased in all septic mice with levels up to 44-fold those of healthy control mice (*p* < 0.0001 for all time cohorts) (Fig. 2b).

During acute (1-day sepsis group) and subacute (3-day and 5-day sepsis groups) sepsis, plasma ACTH concentrations were similar to those of healthy control mice (all *p* > 0.05) (Fig. 2a). In prolonged septic mice (7-day sepsis group), plasma ACTH concentrations were lower than those of healthy control mice (*p* = 0.01) (Fig. 2a). In contrast, plasma concentrations of POMC were higher in septic mice than in healthy control mice (*p* = 0.05) (Fig. 2c).

Hepatic 5α-reductase mRNA was suppressed throughout the course of sepsis-induced critical illness (all *p* < 0.05 vs. healthy control mice), whereas 5β-reductase mRNA was only suppressed during the acute phase (*p* < 0.05 for 1-day sepsis group vs. healthy control mice) (Fig. 2d).

#### Hypothalamic paraventricular mRNA expression of CRH and AVP in acute and prolonged sepsis-induced critical illness

**Fig. 3:**
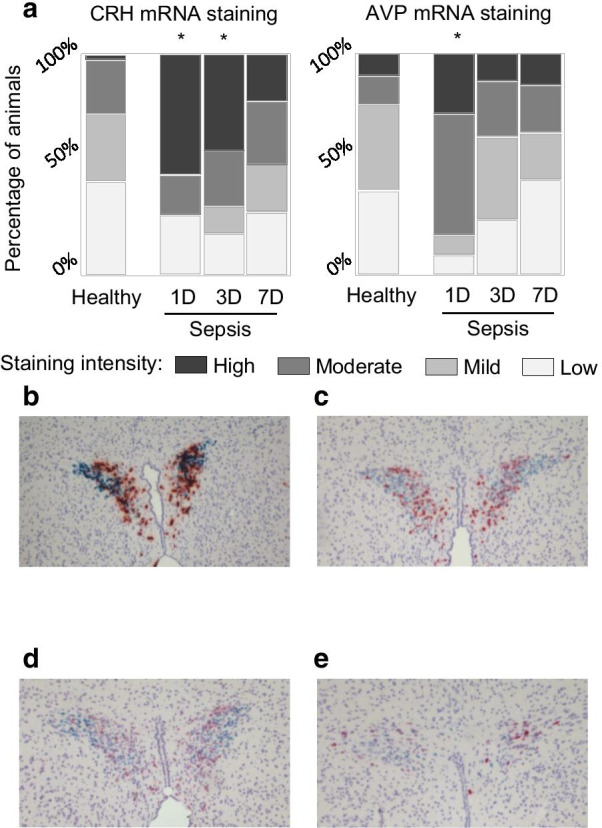
CRH and AVP mRNA expression in the hypothalamic paraventricular nucleus. **a** Semiquantitative scoring of CRH mRNA expression (left panel) and AVP mRNA expression (right panel). Data are represented as cumulative percentages of the respective group. *p < 0.05 as compared to healthy control mice. **b**–**e** Representative examples of in situ* hybridization* stained cross-hypothalamic sections scored high (**b**), moderate (**c**), mild (**d**) and low (**e**) with blue staining for CRH and red staining for AVP. D, days

In acute sepsis-induced critically ill mice (1-day sepsis group), paraventricular mRNA expression of both CRH and AVP was increased as compared with healthy controls (*p* = 0.04 and *p* = 0.03, respectively) (Fig. 2). Septic mice with an illness duration of 3 days still had increased expression of CRH (*p* = 0.03), but not of AVP (*p* = 0.52) (Fig. 3). In prolonged septic mice (7-day sepsis group), mRNA expression of CRH and AVP was comparable to those of healthy control mice (both *p* > 0.05) (Fig. 3).

#### Pituitary expression of POMC and ACTH and transcriptional regulation of processing and secretion hereof during acute and prolonged sepsis-induced critical illness

**Fig. 4:**
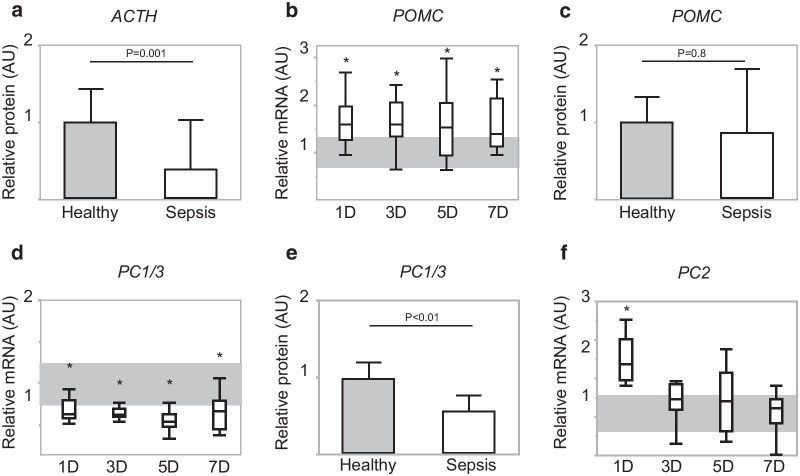
Pituitary expression of POMC and ACTH and of the processing enzymes PC1/3 and PC2. **a** Relative pituitary protein expression of ACTH. **b** Relative pituitary gene expression of POMC. **c** Relative pituitary protein expression of POMC. **d** Relative pituitary gene expression of PC1/3. **e** Relative pituitary protein expression of PC1/3. **f** Relative pituitary gene expression of PC2. All bars and whiskers represent median and interquartile range. For panels **a**, **c** and **e**, healthy controls are represented as box-and-whisker plots. For panels **b** and **d**, shaded gray area represents average interquartile range of the healthy controls over all days. *p ≤ 0.05 compared to the healthy control mice. AU, arbitrary units; D, days

Pituitary protein content of mature ACTH was lower in septic mice than in healthy controls (*p* = 0.001), all time cohorts combined (Fig. 4a). In contrast, of the ACTH precursor hormone POMC, the gene expression was higher than normal irrespective of duration of illness (*p* < 0.05 for all time cohorts) (Fig. 4b), whereas the protein content was normal (*p* = 0.8) (Fig. 4c). PC1/3, responsible for proteolytic cleavage and processing of POMC into ACTH, was acutely and persistently downregulated at both the gene and the protein level during sepsis-induced critical illness (all *p* < 0.01) (Fig. 4d, e). Gene expression of proprotein convertase 2 (PC2), mediating further processing of ACTH into shorter fragments, was increased during the acute phase of sepsis (*p* = 0.02), but not altered during the subacute and prolonged phase of sepsis-induced critical illness.

**Fig. 5:**
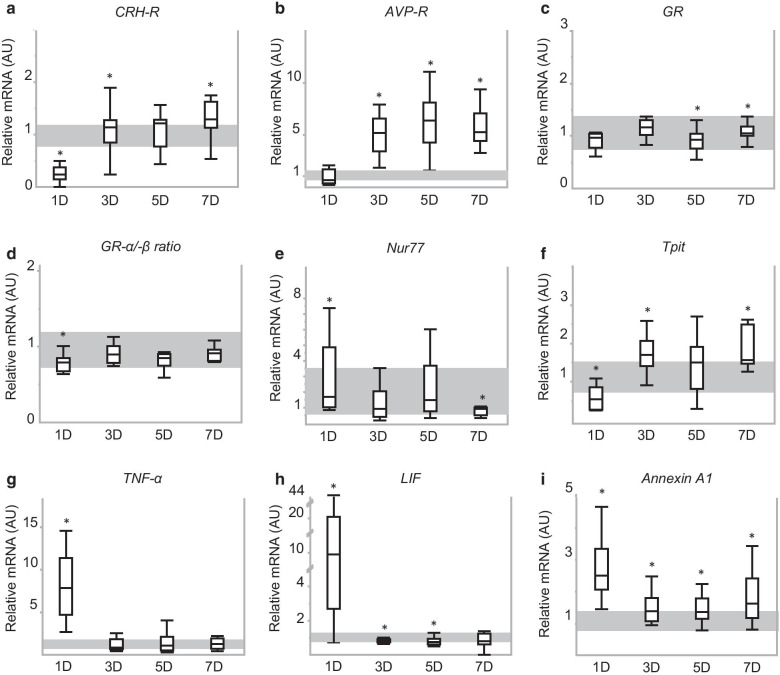
Expression of mediators of POMC or PC1/3 expression. **a**–**i** Relative pituitary gene expression of receptors and transcription factors known to regulate POMC and PC1/3 expression: CRH-R (**a**), AVP-R (**b**), GR (**c**), GRα/β ratio (**d**), Nur77 (**e**), Tpit (**f**), TNF-α (**g**), LIF (**h**) and Annexin A1 (**i**). Bars and whiskers represent median and interquartile range. Shaded gray area represents interquartile range of the controls. *p ≤ 0.05 compared to the healthy control mice. AU, arbitrary units; D, days

Gene expression of the CRH-R, regulator of POMC expression was acutely suppressed (*p* < 0.0001 for 1-day sepsis group) but increased above normal levels during both subacute and prolonged sepsis (3-day and 7-day sepsis groups, all *p* < 0.05) (Fig. 5a). Gene expression of the AVP-R, through which signaling is assumed to potentiate CRH-induced POMC expression, did not differ between acute septic mice (1-day sepsis group) and healthy controls (*p* = 0.2), but increased substantially during the subacute and prolonged phase of sepsis (3-day, 5-day and 7-day groups, all *p* < 0.0001) (Fig. 5b). Gene expression of total GR, through which signaling is known to regulate both POMC and PC1/3, was lower than normal in mice with a longer duration of critical illness (5-day and 7-day sepsis groups, both *p* < 0.05) (Fig. 5c), whereas the ratio between GRα and GRβ expression was lower than normal only in acute critically ill mice (1-day sepsis group, *p* = 0.05) (Fig. 5d).

Gene expression of nerve growth factor IB, also called Nur77, was increased during acute sepsis-induced critical illness (*p* = 0.01 for 1-day sepsis group), unaltered during the subacute phase (*p* = 0.6 for 3-day and 5-day sepsis groups) and decreased during prolonged phase (*p* = 0.001 for 7-day sepsis group) (Fig. 5e). Gene expression of Tpit (or TBX19), a Nur77-synergistic POMC-activating transcription factor, was decreased during the acute phase (*p* = 0.002 for 1-day sepsis group) and increased during the subacute (*p* = 0.02 for 3-day sepsis group) and prolonged phase of sepsis-induced critical illness (*p* < 0.001 for 7-day sepsis group) (Fig. 5f).

Pituitary gene expression levels of pro-inflammatory cytokines TNF-α and LIF were higher than normal, but only in the acute phase of sepsis-induced critical illness (1-day sepsis group) and this by more than sevenfold and eightfold, respectively (Fig. 5g, h). During the subacute (3-day and 5-day sepsis groups) and prolonged phase (7-day sepsis group), pituitary TNF-α mRNA levels were similar to those of healthy control mice (Fig. 5g). Pituitary gene expression of LIF was even lower than normal during the subacute phase of sepsis-induced critical illness (3-day and 5-day sepsis groups) (Fig. 5h). Annexin A1 mRNA levels were increased during all phases of sepsis-induced critical illness (all *p* < 0.05 versus healthy control mice, Fig. 5i).

#### Adrenocortical architecture and markers of adrenocortical steroidogenesis during acute and prolonged sepsis-induced critical illness

**Fig. 6:**
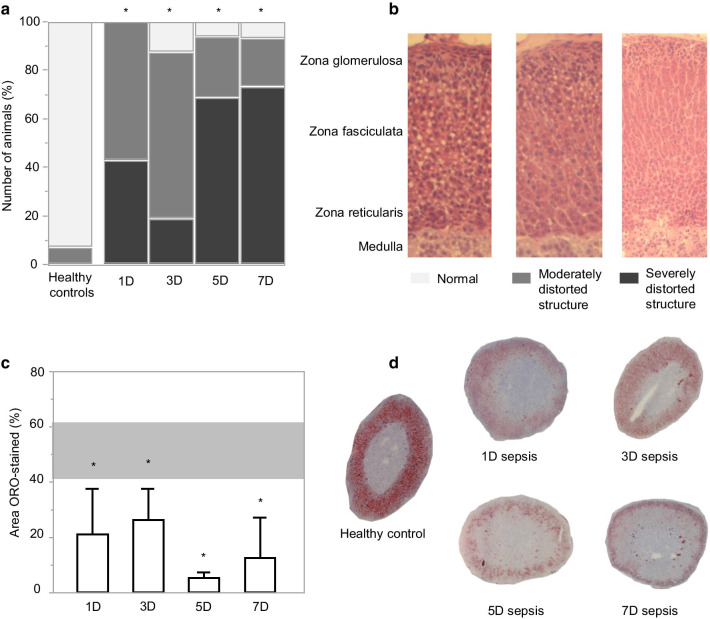
Adrenocortical architecture and adrenal cholesterol ester storage. **a** Semiquantitative scoring of adrenal architecture. Data are represented as cumulative percentages of the respective group. **b** Representative examples of HE stained images for each score. **c** Adrenal cholesterol ester storage quantification. Bars and whiskers represent median and 75th quartile percentage of ORO-stained area relative to total adrenal area. Horizontal gray box represents the interquartile range of the healthy controls. **d** Representative examples of ORO-stained sections of the healthy groups, acute phase (1-day sepsis group), subacute phases (3- and 5-day sepsis group) and prolonged phase of critical illness (7-day sepsis group). *p < 0.05 as compared to the respective healthy control group. D, days

Microscopic semiquantitative scoring unveiled loss of normal, radial cord-like architecture of the *zona fasciculata* in the adrenal cortex of all septic mice as compared with healthy controls (*p* < 0.0001) (Fig. 6a, b). Such loss of adrenocortical architecture was more pronounced (i.e., worse scores) in prolonged sepsis-induced critically ill mice (5-day and 7-day sepsis groups) as compared with critically ill mice with an illness duration of only 3 days (*p* < 0.01 for both comparisons). Also, adrenocortical cholesterol staining was lower than normal in all critically ill mice (all *p* < 0.05) (Fig. 6c, d).

**Fig. 7:**
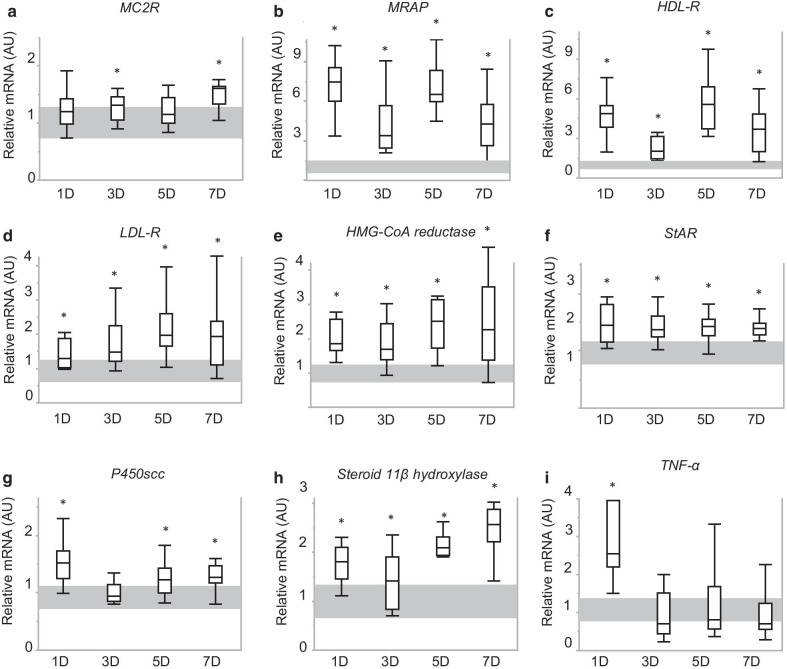
Expression of markers and regulators of adrenal steroidogenesis. **a**–**i** Relative adrenal gene expression of markers and regulators of adrenal steroidogenesis: MC2R (**a**), MRAP (**b**), HDL-R (**c**), LDL-R (**d**), HMG-CoA reductase (**e**), StAR (**f**), P450scc (**g**), steroid 11β dehydrogenase (**h**) and TNF-α (**i**). Bars and whiskers represent median and interquartile range. Shaded gray area represents interquartile range of the controls. *p ≤ 0.05 compared to the healthy control group mice. AU, arbitrary units; D, days

Adrenal gene expression levels of the ACTH-receptor MC2-R were either increased (*p* = 0.02 for 3-day sepsis group and *p* < 0.0001 for 7-day sepsis group), or normal (*p* = 0.06 for 1-day sepsis group and *p* = 0.06 for 5-day sepsis group) (Fig. 7a). Gene expression of MRAP was substantially increased from 1 day of sepsis onwards (*p* < 0.0001 for all sepsis groups versus healthy control mice) (Fig. 7b). Markers of adrenal uptake and synthesis of cholesterol (HDL-R and LDL-R, HMG-CoA reductase, StAR) were all elevated during all phases of sepsis-induced critical illness (*p* < 0.01 for all sepsis groups) (Fig. 7c–f). Gene expression of P450scc, converting cholesterol to pregnenolone, was increased during acute, subacute and prolonged critical illness (all *p* < 0.01 versus healthy control mice) (Fig. 7g). Gene expression of steroid 11β-hydroxylase, catalyzing 11 deoxycorticosterone into corticosterone, was also upregulated at the mRNA level during all phases of critical illness (*p* < 0.01 for all time cohorts) (Fig. 7h). Similar to the observations in the pituitary gland, mRNA levels of the pro-inflammatory cytokine TNF-α were higher than normal only in the acute phase of sepsis-induced critical illness (1-day group) (*p* < 0.0001) (Fig. 7i).

## Discussion

The two studies of human patients suffering from sepsis revealed that, in the face of the known ACTH–cortisol dissociation, plasma concentrations of the ACTH precursor POMC were substantially elevated from the acute into the prolonged phase of sepsis-induced critical illness. In the mouse model of sepsis, this hormonal phenotype was confirmed. In the mice, sepsis was found to acutely, though transiently, increase hypothalamic expression of CRH and AVP, followed by an upregulation of both CRH and AVP receptor expression at the pituitary level. Also, from acute throughout prolonged sepsis, pituitary POMC gene expression was elevated. Together, these findings are suggestive of a centrally activated HPA axis irrespective of illness duration. In contrast, markers of processing POMC into ACTH and of ACTH secretion, known to be negatively regulated by glucocorticoid receptor ligand binding, were suppressed at all time points, offering explanation for the low ACTH and the high POMC plasma levels. Although adrenocortical structure was distorted, markers of adrenocortical steroidogenic activity were increased. The possibility that the latter is driven by high circulating POMC requires further investigation.

The first important and novel finding was the high levels of circulating POMC in both the acute and prolonged human sepsis studies as well as in the mouse model of sepsis. This observation corroborates a hypothalamic activation in response to sepsis. The mouse study indeed revealed that hypothalamic activators of POMC expression, CRH and AVP, were ubiquitously expressed, their pituitary receptors were upregulated, and pituitary levels of POMC gene expression were high. The preserved CRH and AVP expression is in line with a previous study of experimental septic shock in rats and of human septic shock non-survivors [19], in which molecules drive the preserved CRH and AVP expression remains speculative, but could involve inducible nitric oxide synthase (iNOS) [19, 20], cytokines [21] and catecholamines [22]. These data could all point to a centrally activated cortisol production, were it not that pituitary ACTH levels were low, and circulating levels never increased. Preserved pituitary POMC expression coinciding with reduced pituitary ACTH and normal to low circulating ACTH suggests impaired pituitary processing of POMC into ACTH and secretion hereof in the systemic circulation. Indeed, gene and protein expression of prohormone convertase 1/3, the dominant GR-regulated enzyme responsible for proteolytic cleavage of POMC [8], was suppressed during all phases of sepsis-induced critical illness. In addition, Annexin A1, a GR-regulated potent inhibitor of pituitary exocytosis of stored ACTH [7], was upregulated at the mRNA level.

The differentially altered pituitary gene expression profile of two POMC-activating transcription factors, Nur77 and Tpit, of which the first is positively regulated by CRH-R ligand binding and negatively by GR ligand binding [23] and the second is positively regulated by CRH-R ligand binding but not affected by GR ligand binding [24], confirms the presence of both stimulating CRH and AVP signaling and suppressing GR-ligand-binding-induced signaling, during sepsis. Such centrally stimulated ongoing POMC gene expression together with suppressed downstream processing into ACTH could result in an increased availability of POMC protein. This POMC can leach out from the corticotrope cells via the constitutive secretory pathway, bringing about the high circulating levels [7, 25]. Such POMC leaching into the circulation could explain why POMC protein does not appear to accumulate in the pituitary gland despite increased POMC gene expression and impaired POMC processing into ACTH.

Whether increased circulating POMC levels are solely explained by increased CORT-induced feedback inhibition at the level of the pituitary GR, in the presence of ongoing central activation by CRH/AVP, remains unknown. The surprising finding that in human sepsis patients, both ACTH and cortisol rose upon recovery, suggests that the driving GR-binding ligands may be eliminated upon recovery. Hence, these GR-binding ligands could be distinct from glucocorticoids, as cortisol was found to be even higher during recovery than during the prolonged ICU phase [10], in which other molecules that activate the GR in the context of sepsis-induced critical illness remain currently unknown. However, bile acids, of which the circulating levels are known to rise in this condition [26] and to normalize upon recovery, could be one of other possible candidates [27, 28].

In the adrenal cortex, gene expression of regulators and markers of steroidogenesis, which are assumed to be predominantly ACTH stimulated, was found to be upregulated in the absence of increased plasma ACTH. One possible explanation could be an increased ACTH sensitivity [29], a possibility that is supported by the here observed increased expression of MRAP, a facilitator of MC2-R expression and signaling [30], coinciding with normal or increased expression of the ACTH receptor MC2-R itself. However, in patients with sepsis, ACTH responses to cosyntropin are never elevated as shown previously [10], not supporting this hypothesis. Alternatively, the MC2-R could be activated by ligands other than ACTH. In theory, small ACTH fragments, not detected by highly specific immunoenzymometric assays, could exert such steroidogenic effects. However, small ACTH fragments have previously shown to be absent in septic and non-septic ICU patients [9]. In addition, pituitary expression of PC2, which could have increased further processing to smaller fragments, was increased only in the acute phase of sepsis. Alternatively, increased circulating POMC could play such a role and contribute to steroidogenesis. Such a steroidogenic role for POMC has already been suggested by cases of clinically overt Cushing’s syndrome revealing high plasma concentrations of POMC and (very) low plasma ACTH [12, 13]. If POMC can drive steroidogenesis, this could be either a direct effect through binding of POMC to MC2-R or an indirect effect if POMC is locally cleaved into ACTH at the adrenocortical level [13]. Whether such a POMC effect contributes to the increased mRNA of the steroidogenic enzymes that we found in the septic mice, and thus to the elevated plasma CORT while ACTH is not increased and while the adrenocortical structure and cholesterol ester storage was compromised, remains to be investigated. Indeed, the observed contrast between the increased mRNA levels of the steroidogenic enzymes, particularly those involved in cholesterol supply (HDL-R, LDL-R and HMG-CoA reductase) and the pronounced lipid depletion in the adrenal cortex, is striking. Whether the upregulation of these enzymes at the mRNA level without effectively resulting in augmented adrenocortical cholesterol content is the consequence of low circulating cholesterol, is unknown. Alternatively, a local inflammation-driven steroidogenesis or increased sympathetic activation in response to sepsis has been suggested to explain high systemic CORT in the face of low plasma ACTH [31, 32]. However, we here report only a transient rise in adrenal TNF-α mRNA with suppressed levels present in the prolonged phase of illness, not supporting a local inflammatory process driving adrenocortical steroidogenesis.

This study has some limitations. First, in the human studies, plasma hormone concentrations were compared between septic patients and matched healthy subjects; a descriptive analysis only and the impact of frequently used drugs, baseline BMI and nutritional status or other potential confounders were not investigated. Second, the mouse model of sepsis-induced, antibiotics-treated and fluid-resuscitated critical illness is a model of fecal peritonitis not treated surgically, unlike what would be done for human patients. Third, with the chosen experimental design, we could not provide insights in alterations occurring prior to the 1-day time point. Fourth, the interpretation of the data as suggestive for suppressed POMC processing into ACTH during critical illness was based only on gene and protein expression and not on quantification of enzyme activity. Fifth, because of the small size of a mouse pituitary, we used whole pituitary homogenates for gene and protein expression and because of a limited amount of proteins yielded from pituitary homogenates, it was not possible to perform western blot analysis for all transcriptional regulators. Finally, the scarce knowledge on any potential steroidogenic capacity of POMC requires further research.

## Conclusion

The findings of these studies are compatible with glucocorticoid-receptor-ligand-binding-induced inhibition of pituitary processing of POMC into ACTH and with preserved CRH/AVP-driven expression of POMC, resulting in elevated circulating POMC levels in sepsis. Whether high circulating POMC exerts adrenocortical steroidogenic activity requires further investigation.

## Supplementary Information

**Additional file 1.** Additional file 1 contains supplemental Materials and Methods, supplemental table 1 with the list of used probes and primers for gene expression analysis and supplemental table 2 with the list of used antibodies for protein expression analysis.

## Data Availability

Some or all datasets generated during and/or analyzed during the current study are not publicly available but are available from the corresponding author on reasonable request.

## Abbreviations

ACTH: Adrenocorticotropic hormone
APACHE II: Acute Physiology and Chronic Health Evaluation II
AVP: Vasopressin
BMI: Body mass index
CRH: Corticotropin-releasing hormone
GR: Glucocorticoid receptor
HDL(-R): High-density lipoprotein (receptor)
HES: Hydroxyethyl starch
HMG-CoA: 3-Hydroxy-3-methylglutaryl coenzyme A reductase
ICU: Intensive care unit
LDL(-R): Low-density lipoprotein (receptor)
LD: Last day
LIF: Leukemia inhibitory factor
MC2-R: Melanocortin receptor 2
MRAP: Melanocortin receptor 2 accessory protein
ORO: Oil-Red-O
POMC: Pro-opiomelanocortin
(m)RNA: (Messenger) ribonucleic acid
StAR: Steroidogenic acute regulatory protein
TNF-α: Tumor necrosis factor alpha
