# Supplementary material for: The role of pro-opiomelanocortin in the ACTH–cortisol dissociation of sepsis
Source: Crit Care. 2021 Feb 16;25:65. doi: 10.1186/s13054-021-03475-y (PMC7885358; doi:10.1186/s13054-021-03475-y)
Supplement: Supplementary file 1 — Additional file 1. Additional file 1 contains supplemental Materials and Methods, supplemental table 1 with the list of used probes and primers for gene expression analysis and supplemental table 2 with the list of used antibodies for protein expression analysis. [file 13054_2021_3475_MOESM1_ESM.docx]

**The role of pro-opiomelanocortin in the ACTH-cortisol dissociation of sepsis**

**Additional file 1**

Arno Téblick^1^, Sarah Vander Perre^1^, Lies Pauwels^1^, Sarah Derde^1^, Pieter Wouters^1^, Tim Van Oudenhoven^1^, Lies Langouche^1^, Greet Van den Berghe^1^

^1^ Clinical division and Laboratory of Intensive Care Medicine, Department of Cellular and Molecular Medicine, KU Leuven, Leuven, Belgium

**Corresponding author**: Greet Van den Berghe. E-mail: greet.vandenberghe@kuleuven.be

**Keywords**: Sepsis, pituitary, adrenal, adrenocorticotropic hormone, pro-opiomelanocortin, glucocorticoid receptor

**Declaration of interest and funding**: All authors declare that there is no conflict of interest. This work was supported by the Research Foundation Flanders (FWO) grant G091918N to GVdB, the European Research Council Advanced Grant (AdvG-2017-785806 to GVdB) from European Union’s Horizon 2020 research and innovation programme and the Methusalem programme of the Flemish Government (METH/14/06 to GVdB and LL via the KU Leuven).

**Table of content:**

Supplemental methods

Additional file 1: Table S1. List of used probes and primers.

Additional file 1: Table S2. List of used antibodies.

Supplemental methods

*Human studies of critically ill patients with sepsis*

Two human studies were performed to document the plasma POMC concentration time course during sepsis-induced critical illness and in the recovery phase, in relation to ACTH and cortisol. Human study 1 focused on ICU patients during the first week in ICU. We selected, from a previous study (1), all available patients who suffered from sepsis at study inclusion (n=51), according to the 1992 Sepsis-2 criteria (2), and who did not meet exclusion criteria and 20 demographically matched overnight-fasted healthy controls. Exclusions criteria were pre-admission risks for HPA axis dysfunction, which comprised chronic treatment with glucocorticoids, or anti-steroid chemotherapy within the last 3 months, steroid treatment in the hours preceding ICU admission (e.g. during surgery or in the emergency room) or other drugs predisposing to adrenal insufficiency (phenytoin, rifampicin, glitazones, imipramine, phenothiazine, phenobarbital). In this study, plasma samples were collected daily throughout the first week in ICU. Human study 2 focused on the prolonged phase of critical illness, beyond 1 week in the ICU until recovery on a regular ward (3). From this study, all available patients who suffered from sepsis at inclusion (n= 45) with an ICU stay of at least 4 weeks and 20 demographically matched healthy controls were included, with similar exclusion criteria as for human study 1. In this study, plasma samples were collected weekly, beyond the first week in ICU until ICU day 49 and 1 week after ICU discharge. Baseline characteristics are described in Table 1. Data from patients who died or were discharged before day 7 in study 1 or before day 49 in study 2, were included until time of discharge or death. Total plasma cortisol concentrations were quantified with use of RIA (Immunotech, intra-assay coefficient of variation of 5.8%, inter-assay coefficient of variation of 9.2%, analytical sensitivity 0.36 µg/dl), plasma ACTH concentrations were measured by a highly sensitive and specific double-monoclonal immunoradiometric assay (Brahms Diagnostics, intra-assay coefficient of variation of 1.3% - 7.9%, inter-assay coefficient of variation of 4.4% - 16.2%, analytical sensitivity 1.2 pg/ml) and plasma POMC concentrations were quantified by a specific sandwich enzyme-linked immunosorbent assay (MyBioSource Inc.,intra-assay and inter-assay coefficient of variation of <10%, analytical sensitivity 0.1 ng/ml) without cross-reactivity with ACTH or other POMC derivatives. Written informed consent was obtained from all patients and their next of kin and from all healthy volunteers. The study protocols and consent forms were approved by the Institutional Ethical Review Board (ML4190 and ML11107).

*Mouse model of sepsis*

To study the impact of sepsis on hormonal alterations and possibly involved pathways in the liver, the hypothalamus and the pituitary and the relationship hereof with abnormalities in the adrenal cortex, male, 24-week old C57BL/6J mice (mature adult age) (Janvier SAS) were randomly allocated to a ‘sepsis’ or a ‘healthy control’ group. To assess the impact of duration of sepsis, both the ‘sepsis’ and ‘healthy control’ group were subdivided into 4 time-cohort groups with increasing duration of illness (1-day, 3-days, 5-days or 7-days between randomization and sacrifice). In the ‘sepsis groups’, sepsis was induced by standardized cecal-ligation and puncture, which results in tissue trauma, ischemia of the ligated cecum, and abdominal polymicrobial sepsis (4). Mice were anesthetized with intraperitoneal ketamine/xylazine and received oxygen during the entire procedure. First, the left internal jugular vein was cannulated with a catheter during surgical exploration of the neck region and subsequently tunneled to the back to allow continuous perfusion but still provide free movement of the animal during the study period. Next, a median laparotomy was performed, followed by ligation (50%) and single trough-and-through puncture (with a 18-gauge needle) of the caecum. After closure of both peritoneum and skin with a 5-0 suture, mice were transferred to individual cages and intravenous fluid therapy was started. During the first 24h mice were resuscitated with a 4/1 crystalloid/colloid mixture (Plasmalyte, Baxter) and hydroxyethyl starch (HES) 6% at an infusion rate of 0.3 ml/hour. Hereafter, septic mice allocated to the 3-days, 5-days or 7-days sepsis groups received intravenous parenteral nutrition (Oliclinomel N7E, Baxter), rated at 0.2 ml/h. As such the total daily intake of calories approximated 5.8 kcal/d (~45% of normal daily intake of healthy mice), mimicking critical illness-induced lack of feeding. Septic mice further received twice daily a subcutaneous injection with broad-spectrum antibiotics (Imipenem/Cilastin (Aurobindo Pharma)) and opioid-analgetics (Buprenophine (Vetergesic)) with the initial bolus 6 hours after randomization. *A priori* defined exclusion criteria were death during the procedure or death during the study period and a dysfunctional or clogged intravenous catheter. Animals randomized to the ‘healthy control group’, were transferred to individual cages and received *ad libitum* standard chow (ssnif R/M-H, ssniff Spezialdiäten GmbH) and tap water throughout the study period. All animal cages were kept in an animal cabinet under controlled temperature (27°C) and 12 hours light and dark cycles. Healthy animals did not undergo any invasive procedure and did not receive antibiotics or opioid-analgetics. At the end of the study period, mice received intraperitoneal ketamine/xylazine and were sacrificed with terminal cardiac puncture. The deceased animal was decapitated. One operator extracted the whole brain from the skull, after which it was immediately frozen on dry ice. Then, the exposed pituitary was carefully dissected from the *sella turcica* and snapfrozen in liquid nitrogen and stored at -80°C until further processing. Meanwhile, operator 2 extracted the liver and both left and right adrenal gland. The liver and left adrenal gland were snapfrozen in liquid nitrogen and stored at -80°C (for gene and protein expression analysis), the right adrenal gland was frozen in Tissue-Tek (Sakura Finetek) (for histological analysis). All tissues were collected, frozen and stored within 10 minutes after the end of cardiac puncture (maximal warm ischemia time). The study was continued until 15 surviving animals per study group and per time cohort were reached. Total number of animals per group at the end of the study is as follows : Healthy 1-day: n=15, Healthy 3-days: n=15, Healthy 5-days: n=15, Healthy 7-days: n=16, Sepsis 1-day: n=15, Sepsis 3-days: n=16, Sepsis 5-days: n=16, Sepsis 7-days: n=15. This sample size was based on an estimated effect size of 50% increase in plasma corticosterone (CORT), a 15% reduction in CRH expression and a 50% reduction in adrenal lipid content, to be detected with an α-error ≤0.05 and >80% power. All animals were treated according to the Principles of Laboratory Animal Care (U.S. National Society of Medical Research) and to the European Union Directive 2010/63/EU concerning the welfare of laboratory animals. The study was approved by the Institutional Ethical Committee for Animal Experimentation (P134-2013) and complied with the essential 10 ARRIVE guidelines (5).

*Plasma analyses of HPA hormones and binding proteins*

To measure plasma concentrations of hormones and proteins, whole blood was sampled during sacrifice by cardiac puncture and immediately centrifuged (6000 rpm, 10 min at 4°C). Plasma was stored at -80°C until analysis. Plasma concentrations of CORT, ACTH and POMC were quantified using commercially available enzyme-linked immunosorbent assays kits (DRG). Cortisol-binding globulin (CBG) was quantified by western blot (anti-CBG antibody (ID: 191053), Abcam) as previously documented (6). Plasma albumin was measured with a colorimetric assay (Thermo Scientific). As free CORT measurements were not feasible, levels of free plasma CORT were estimated based on plasma total cortisol, CBG and albumin concentrations with the following calculation [relative plasma total corticosterone – ((0.85*relative plasma CBG*relative plasma total corticosterone)+(0.10*relative plasma albumin*relative plasma total corticosterone))]. This formula has been based on the physiological *in vivo* percentage distribution of CORT (7-10). Data are presented as fold-difference as compared with the median of the day-1 healthy control mice (arbitrary unit, AU).

*Hypothalamic CRH and AVP expression with in situ hybridization*

We performed chromogenic RNAscope *in* situ hybridization (Advanced Cell Diagnostics) to quantify gene expression of CRH and AVP in the paraventricular nucleus (PVN) of the hypothalamus. Whole brain tissue was fixed in 10% neutral formalin buffer and embedded in paraffin using standard procedures. Next, 5 µm-thick coronal sections were cut onto Superfrost Plus slides and stored at room temperature. RNAscope 2.5 HD Duplex Assay was performed as per the manufacturer’s instructions (11) on slides containing a brain section through the PVN. Following RNAscope probes were used: CRH (ID: 316091) in the blue channel and AVP (ID: 401391) in the red channel. Images were captured at 40x magnification with standardized parameters using a Leica DM3000 bright-field microscope. Intensities of CRH and of AVP were both scored semi-quantitatively by two independent investigators, blinded for randomization. Only AVP stained in the region of the parvocellular neurons was taken into account for semi-quantitatively scoring as AVP expressed in other brain regions is not involved in the regulation of the pituitary corticotropes. Any discrepancy was resolved by consensus. A 4-level score system was defined as: ‘0’ for absent to low staining, ‘1’ low to moderate staining, ‘2’ high staining and ‘3’ very high staining to saturation of the region of interest.

*RNA isolation and reverse transcription polymerase chain reaction analysis*

We quantified gene expression of 1) pituitary POMC, the major cleavage-enzyme proprotein convertase 1 (PC1/3) and the transcriptional regulators hereof (12, 13) and proprotein convertase 2, 2) hepatic CORT-metabolizing enzymes and 3) adrenal key regulators and markers of adrenal steroidogenesis. Total RNA was isolated from snap frozen pituitary and adrenal tissue with the NucleoSpin RNA/protein isolation kit (Machery-Nagel, Düren, Germany) and from snap frozen liver tissue with RNeasy isolation kit (Qiagen). Genomic DNA was removed with DNAse treatment. Total RNA (500 ng) was reverse-transcribed with use of random hexamers (Invitrogen). Commercial TaqMan probes (Applied Biosystems) (Supplemental table 1a and b) and customized forward/reverse primers (supplemental table 1c) were used for real-time gene expression. Relative gene expressions were calculated with the 2-∆ΔCT method with 18S ribosomal RNA (Rn18s) selected as housekeeping gene.

*Protein isolation and immunoblotting*

We quantified pituitary protein content of the hormones POMC and ACTH and of the enzyme PC1/3. Protein isolation from a whole pituitary homogenate was performed with the NucleoSpin RNA/Protein kit (Machery-Nagel, Düren, Germany). After isolation, protein concentration was calculated with the Protein Assay Reagent kit (Pierce Biotechnology Inc.). Twenty (20) µg protein was diluted in Laemmli buffer ((2-mercaptoethanol (0,1%), Tris-HCl (63mM, pH 6.8), bromophenol blue (0,0005%), glycerol (10%) and sodium dodecyl sulfate (2%)). To quantify PC1/3 protein expression, proteins were separated by SDS-PAGE on a 4-20% gel and subsequently electroblotted on a nitrocellulose membrane (90min at 70V), to quantify POMC and ACTH protein expression, proteins were separated by SDS-PAGE on a 16.5% gel and subsequently electroblotted on a PVDF membrane (60min at 70V). Membranes were blocked with Tris buffered saline containing 1% Tween 20%, and 5% Bovine Serum Albumin and incubated overnight at 4°C with commercially available primary antibodies (supplemental table 2). Secondary antibodies were purchased from DakoCytomation. Blots were visualized with the G:BOX XRQ (SynGene) and bands were quantified with Syngene software.

*Structural integrity and quantification of cholesterol ester content of the adrenal cortex*

Whole adrenal glands were frozen in Tissue-Tek (Sakura Finetek) and cut at 8 µm thickness with a microtome-cryostat and serially collected on Superfrost Plus slides. Structural integrity of the adrenal cortex was evaluated on hematoxylin and eosin stained serial tissue sections of the adrenal gland and subsequently scored semi-quantitatively by two independent investigators, blinded for randomization. Any discrepancy was resolved by consensus. A ‘2’ score was given when the three adrenocortical zones were clearly distinguishable and the fasciculate zone had a normal, radial, cord-like architectural pattern; a ‘1’ score was given when there was a moderate distortion of the adrenocortical zones with or without the presence of tissue edema; a ‘0’ score was given when the three adrenocortical zones were severely distorted with the presence of extensive tissue damage. Adrenocortical cholesterol esters storage was quantified on Oil-Red-O (ORO) stained serial tissue sections. Sections were dried at room temperature, fixed in propylene glycol, and stained overnight in 0.5% ORO (Sigma-Aldrich) in propylene glycol and counterstained with hematoxylin. ORO stained sections were analyzed for the relative amount of redness in the adrenal cortex with ImageJ 1.52a.

*Statistical analysis*

Data are presented as box plots with median, interquartile range (25^th^ - 75^th^ percentiles) and 10^th^ and 90^th^ percentiles or as mean and standard error of the mean (SEM). Differences between groups were analyzed with use of Mann-Whitney U, Chi-squared or Fisher exact test, as appropriate. Time series data from both human studies were analyzed with use of repeated-measures ANOVA, after transformation of the results to obtain a normal distribution. A two-sided *p* value equal or less than 0.05 was considered statistically significant. No corrections for multiple comparisons were performed. All statistical analyses were done with JMP pro 14 (SAS Institute Inc.).

Additional file 1: Table S1. List of used genes.

a) Pituitary gene expression analysis (Taqman probes)

| Gene symbol (mus musculus) | Common name | Product name (ThermoFisher Scientific) |
| --- | --- | --- |
| *Pomc* | Pro-opiomelanocortin (POMC) | Mm00435874_m1 |
| *Crhr1* | CRH-receptor 1 | Mm00432670_m1 |
| *Avpr1b* | AVP-receptor 1b | Mm01700416_m1 |
| *Nr3c1* | (Total) glucocorticoid receptor (GR) | Mm00433832_m1 |
| *Nr4a1* | Nur77 | Mm01300401_m1 |
| *Tbx19* | Tpit | Mm00453377_m1 |
| *Tnf* | Tumor Necrosis Factor alpha (TNF-α) | Mm00443258_m1 |
| *Lif* | Leukemia Inhibitory Factor (LIF) | Mm00434762_g1 |
| *Pcsk1* | Proprotein convertase 1 (PC1/3) | Mm00479023_m1 |
| *Pcsk2* | Proprotein convertase 2 (PC2) | Mm01270315_m1 |
| *Anxa1* | Annexin-1 | Mm00440225_m1 |
| *Mc2r* | Melanocortin Receptor 2 | Mm00434865_s1 |
| *Mrap* | Melanocortin Receptor Accessory Protein |  |
| *Scarb1* | HDL-receptor | Mm00450234_m1 |
| *Ldlr* | LDL receptor | Mm01177349_m1 |
| *Hmgcr* | HMG-CoA reductase | Mm01282499_m1 |
| *Star* | StAR | Mm00441558_m1 |
| *Cyp11a1* | Cholesterol side-chain cleavage (P450scc) | Mm00490735_m1 |
| *Cyp11b1* | Steroid 11β-hydroxylase | Mm01204952_m1 |
| *Tnf* | Tumor necrosis factor-α | Mm00443258_m1 |
| *Srd5a1* | 5α-reductase | Mm00614213_m1 |
| *Akr1d1* | 5β-reductase | Mm01165275_m1 |
| *Rn18s* | Ribosomal 18s | Mm03928990_g1 |
| NR3C1 subtypes | Glucocorticoid receptor α  Glucocorticoid receptor β | Primer sequence   - Common forward primer:  AAAGAGCTAGGAAAAGCCATTGTC - Specific reverse primer: TCAGCTAACATCTCTGGGAATTCA   CTGTCTTTGGGCTTTTGAGATAGG |

Additional file 1: Table S2. List of used antibodies

| Protein name (mus musculus) | Common name | Manufacturer and productcode  (Primary antibody) ; band analysed |
| --- | --- | --- |
| POMC | Pro-opiomelanocortin | Abcam (74976) - 31 kDa |
| ACTH | Adrenocorticotropic hormone | Abcam (74976) - 4,5 kDa |
| PCSK1 | Proprotein convertase 1 | Abcam (3532) - 87 kDa |

References (supplement):

1. Meersseman P, Boonen E, Peeters B, Vander Perre S, Wouters PJ, Langouche L, et al. Effect of Early Parenteral Nutrition on the HPA Axis and on Treatment With Corticosteroids in Intensive Care Patients. J Clin Endocrinol Metab. 2015;100(7):2613-20.

2. Bone RC, Balk RA, Cerra FB, Dellinger RP, Fein AM, Knaus WA, et al. Definitions for sepsis and organ failure and guidelines for the use of innovative therapies in sepsis. The ACCP/SCCM Consensus Conference Committee. American College of Chest Physicians/Society of Critical Care Medicine. Chest. 1992;101(6):1644-55.

3. Peeters B, Meersseman P, Vander Perre S, Wouters PJ, Vanmarcke D, Debaveye Y, et al. Adrenocortical function during prolonged critical illness and beyond: a prospective observational study. Intensive Care Med. 2018;44(10):1720-9.

4. Derde S, Thiessen S, Goossens C, Dufour T, Van den Berghe G, Langouche L. Use of a Central Venous Line for Fluids, Drugs and Nutrient Administration in a Mouse Model of Critical Illness. J Vis Exp. 2017(123).

5. Kilkenny C, Browne WJ, Cuthill IC, Emerson M, Altman DG. Improving bioscience research reporting: the ARRIVE guidelines for reporting animal research. PLoS Biol. 2010;8(6):e1000412.

6. Jenniskens M, Weckx R, Dufour T, Vander Perre S, Pauwels L, Derde S, et al. The Hepatic Glucocorticoid Receptor Is Crucial for Cortisol Homeostasis and Sepsis Survival in Humans and Male Mice. Endocrinology. 2018;159(7):2790-802.

7. Keenan DM, Roelfsema F, Veldhuis JD. Endogenous ACTH concentration-dependent drive of pulsatile cortisol secretion in the human. Am J Physiol Endocrinol Metab. 2004;287(4):E652-61.

8. Lewis JG, Bagley CJ, Elder PA, Bachmann AW, Torpy DJ. Plasma free cortisol fraction reflects levels of functioning corticosteroid-binding globulin. Clin Chim Acta. 2005;359(1-2):189-94.

9. Meyer EJ, Nenke MA, Lewis JG, Torpy DJ. Corticosteroid-binding globulin: acute and chronic inflammation. Expert Rev Endocrinol Metab. 2017;12(4):241-51.

10. Hamrahian AH, Oseni TS, Arafah BM. Measurements of serum free cortisol in critically ill patients. N Engl J Med. 2004;350(16):1629-38.

11. Wang F, Flanagan J, Su N, Wang LC, Bui S, Nielson A, et al. RNAscope: a novel in situ RNA analysis platform for formalin-fixed, paraffin-embedded tissues. J Mol Diagn. 2012;14(1):22-9.

12. Harno E, Gali Ramamoorthy T, Coll AP, White A. POMC: The Physiological Power of Hormone Processing. Physiol Rev. 2018;98(4):2381-430.

13. Philips A, Maira M, Mullick A, Chamberland M, Lesage S, Hugo P, et al. Antagonism between Nur77 and glucocorticoid receptor for control of transcription. Mol Cell Biol. 1997;17(10):5952-9.
